# Exercise dependence and muscle dysmorphia in novice and experienced female bodybuilders

**DOI:** 10.1556/JBA.2.2013.4.8

**Published:** 2013-12-13

**Authors:** Bruce D. Hale, Danielle Diehl, Krista Weaver, Michael Briggs

**Affiliations:** ^*^Berks College, Pennsylvania State University, Reading, PA, USA

**Keywords:** exercise dependence, muscle dysmorphia, female bodybuilders

## Abstract

*Background and aims:* Extensive research has shown that male bodybuilders are at high risk for exercise dependence, but few studies have measured these variables in female bodybuilders. Prior research has postulated that muscular dysmorphia was more prevalent in men than women, but several qualitative studies of female bodybuilders have indicated that female bodybuilders show the same body image concerns. Only one study has compared female bodybuilders with control recreational female lifters on eating behaviors, body image, shape pre-occupation, body dissatisfaction, and steroid use. The purpose of this study was to compare exercise dependence and muscle dysmorphia measures between groups of female weight lifters. *Methods:* Seventy-four female lifters were classified into three lifting types (26 expert bodybuilders, 10 or more competitions; 29 novice bodybuilders, 3 or less competitions; and 19 fitness lifters, at least 6 months prior lifting) who each completed a demographic questionnaire, the Exercise Dependence Scale (EDS), the Drive for Thinness scale (DFT) of the Eating Disorder Inventory-2, the Bodybuilding Dependence Scale (BDS), and the Muscle Dysmorphia Inventory (MDI). *Results:* Female bodybuilders scored higher than fitness lifters for EDS Total, BDS Training and Social Dependence, and on Supplement Use, Dietary Behavior, Exercise Dependence, and Size Symmetry scales of the MDI. *Discussion and conclusions:* Female bodybuilders seem to be more at risk for exercise dependence and muscle dysmorphia symptoms than female recreational weight lifters.

## INTRODUCTION

Although medical practitioners agree that the majority of the population in western societies would benefit from more regular exercise as part of a healthier lifestyle, a small percentage of individuals may develop an obsessive approach that can be damaging physiologically, psychologically and socially. Researchers and clinicians have recently reviewed the decades of emerging literature on excessive exercise in weight lifters, and some have concluded that the behaviors are part of an obsessive-compulsive disorder diagnosis (e.g., Pope, Phillips & Olivardia, 2000), while others suggest that the symptoms are part of a body dysmorphia/body image disorder diagnosis (e.g., Lantz, Rhea & Mayhew, 2001; McCreary & Sasse, 2000), and still others have sought to differentiate it from a primary eating disorder (e.g., Hausenblas & Symons Downs, 2002). More recently, Berczik et al. (2012) have argued forcefully that excessive exercise is a type of behavioral addiction. Unfortunately, almost all of the research that has been reviewed to date on addictive anaerobic exercise behavior (Hale & Smith, 2012; Tod & Lavallee, 2010) has involved male bodybuilding and weightlifting samples.

Whereas most western women seem to score high on the Drive for Thinness scale (DFT; Garner's (1991) Eating Disorder Inventory-2) and yearn to be thin and toned (Thompson, Heinberg, Altabe & Tantleff-Dunn, 1999), men in the last three decades are showing increasing scores in the drive for muscularity (e.g., McCreary and Sasse's (2000) Drive for Muscularity Scale). According to researchers, some weight lifters develop muscle dysmorphia (MD), view themselves as too thin, and may feel pressure to gain muscle size and/or strength even though they may actually be quite large and muscular (Tod & Lavallee, 2010). Components of MD include: body image distortion/dissatisfaction, dietary constraints, pharmacological aids, dietary supplements, exercise dependence, physique concealment, and low self-esteem (Muscle Dysmorphia Inventory, MDI; Rhea, Lantz & Cornelius, 2004).

One of these components, exercise dependence (ED), has been defined as “a craving for leisure time physical activity that results in uncontrollable excessive exercise behavior and that manifests in physiological symptoms (e.g., tolerance, withdrawal) and/or psychological symptoms (e.g., anxiety, depression)” (Hausenblas & Symons Downs, 2002, p. 90). It has also been measured by the Exercise Dependence Scale (EDS; Symons Downs, Hausenblas & Nigg, 2004). In the bodybuilding realm, Smith, Hale and Collins (1998) also created and validated the Bodybuilding Dependence Scale (BDS).

Leone, Sedory and Gray (2005) postulated that MD was more prevalent in men than women, but several qualitative studies of female bodybuilders (Bolin, 1992; Guthrie & Castelnuovo, 1992; Klein, 1986, 1992) have stated that female bodybuilders show the same body image concerns, motivation for muscularity, and workout behaviors as males. While extensive reviews of quantitative studies have shown that male bodybuilders are at high risk for ED (Hale & Smith, 2012; Smith & Hale, 2011) and may also suffer from MD (Tod & Lavallee, 2010), few quantitative designs (Smith & Hale, 2004; Goldfield, 2009) have measured these variables in female bodybuilders.

Goldfield (2009) was one of the few studies to compare 20 female bodybuilders with recreational female lifters on eating behaviors, body image, shape pre-occupation, body dissatisfaction, and steroid use. He reported that bodybuilders scored higher on the Bulimia subscale, Drive for Bulk scale (Blouin & Goldfield, 1995), and Drive for Tone scale (Goldfield, 2009). More recently Hale, Roth, DeLong and Briggs (2010) found that male bodybuilders and power lifters were significantly higher than fitness lifters on EDS Total, seven EDS-R scales, and the three BDS scales. No study to date has compared measures of MD and ED between different groups of female weight lifters.

Although the estimates of bodybuilders suffering from ED and MD may be small in western populations, the study of ED and MD in female weight lifters is warranted. This study hypothesized that female bodybuilders would score significantly higher in ED, MD (Hale & Smith, 2012; Smith & Hale, 2011; Tod & Lavallee, 2010), and lower in DFT (Goldfield, 2009) than female fitness lifters.

## METHODS

### Participants

Seventy-four female weight lifters volunteered and were classified (based on Hurst, Hale & Smith, 2000; Smith & Hale, 2004) as 26 “expert bodybuilders” (10 or more bodybuilding competitions), 29 “novice bodybuilders” (three or less competitions), and 19 “fitness lifters” (at least 6 months prior lifting experience). Participants ranged in age from 18–48 years of age. Participants were recruited from a Pennsylvania university fitness center, several Pennsylvania health clubs, and the annual “Arnold Sports Festival” held in 2009 in Columbus, OH. All volunteers read implied informed consent forms before anonymously completing questionnaires; prior approval was obtained by the University's institutional review board.

## MEASURES

### Demographic questionnaire

All participants completed a demographic questionnaire (adapted from Hale et al., 2010) to examine prior lifting history. The questions concerned lifting experience (years lifting), typical frequency per week (weekly frequency), length of typical workout duration (session time), and intensity (light, moderate, or heavy intensity). In addition, a total lifting time per week variable was created by multiplying the weekly frequency and session time. Participants checked a lifter type category (expert bodybuilder, novice bodybuilder, or fitness lifter) based on their type of lifting experience.

### Exercise Dependence Scale

The Exercise Dependence Scale (EDS; Symons Downs et al., 2004) is a 21-item multidimensional questionnaire with 6-choice Likert scale ranging from “Always” to “Never” based on DSM-IV criteria for substance dependence (American Psychiatric Association, 1994). The seven subscales (Tolerance, *r* = .78; Withdrawal Effects, *r* = .90; Continuance, *r* = .90; Lack of Control, *r* = .82; Reductions in Other Activities, *r* = .75; Time, *r* = .86; Intention, *r* = .89) have all shown acceptable scale score reliability; internal consistency for total EDS score for this study was *r* = .92. Participants are categorized by a total score as “exercise dependent”, “non-dependent symptomatic”, or “non-dependent asymptomatic”. Hausenblas and Symons Downs (2002) and Hausenblas and Giacobbi (2004) presented evidence of concurrent validity of the EDS.

### Bodybuilding Dependence Scale

The Bodybuilding Dependence Scale (BDS; Smith et al., 1998) is a 9-item, 7-choice Likert scale (“Strongly Disagree” to “Strongly Agree”) with three dimensions (Social Dependence, Training Dependence, and Mastery Dependence) to measure the degree to which ED is exhibited in weight lifters based on Veale's (1987) biomedical and psychosocial diagnostic criteria. This measure has demonstrated adequate psychometric internal reliability (Cronbach's alpha of .83, .70, and .89, respectively, in this study), construct and concurrent validity (Hurst et al., 2000; Smith & Hale, 2004), and adequate test–retest reliability (Smith & Hale, 2005) for each scale (*r* = .97, .96, and .94, respectively).

### Muscle Dysmorphia Inventory

The Muscle Dysmorphia Inventory (MDI; Rhea et al., 2004) is a 27-item, 6-point Likert scale measuring six subscales of MD: Size Symmetry, Supplement Use, Exercise Dependence, Pharmacological Use, Dietary Behavior, and Physique Concealment. All subscales showed acceptable internal consistency (Cronbach's alpha = 0.84–0.92) in the present study. Significant correlations between the MDI subscales and the BDS's Training Dependence scale (Smith et al., 1998) and the DFT scale of the Eating Disorder Inventory-2 (Garner, 1991) have provided evidence of convergent validity (Rhea et al., 2004).

### Drive for Thinness Scale

The Drive for Thinness Scale of the Eating Disorder Inventory-2 (DFT; Garner, 1991) is a 7-item 6-point Likert sub-scale to assess weight preoccupation. Research supports its validity and reliability (Garner, 1991; *r* = .80 for internal consistency in this study). Hausenblas and Symons Downs (2002) have used the subscale to categorize participants scoring above “14” as having a possible eating disorder and demonstrating signs of “secondary exercise dependence”.

## PROCEDURE

### Data collection and analysis

After gaining permission from each health club facility and competition site, an implied consent form and the questionnaire packet were distributed to each lifting participant. Participants were asked to complete the packet honestly and anonymously; all volunteers completed their packet and placed it in a sealed envelope to assure confidentiality. Participants took about 15–20 minutes to complete each questionnaire packet.

### Single imputation procedure

In one particular set of questionnaires, a question of the BDS was inadvertently omitted. Because the other set of questionnaires did not have the missing question, a single imputation procedure for missing data could be used. In this technique four of the five questions that made up the Social Dependence subscale of the BDS were used to predict the fifth question. Using backwards selection regression methodology, a model was constructed allowing for prediction of the fifth question. Though single imputation can sometimes lead to underestimated standard errors (Little, 1992), rationalization for this approach is based on the assumption that the incomplete data was matched to the complete data with equal lifting types. Since the appropriate matching of observations was used, this single imputation technique would be equivalent to doing a direct replacement (i.e., finding another lifter; Donders, Van der Heijden, Stijnen & Moons, 2006).

### Statistical analysis

One-way ANOVAs were undertaken on the five demographic questions, total EDS score, and the DFT in order to examine potential group differences. One-way MANOVAs were also calculated on the seven scales of the EDS, three scales of the BDS, and the six scales of the MDI with Tukey post hoc tests used for significant univariate findings to further examine any possible group differences.

### Ethics

All volunteers read implied informed consent forms before anonymously completing questionnaires; prior approval was obtained by the University's institutional review board.

## RESULTS

### Demographic variables

No significant differences occurred in total lifting time between expert and novice bodybuilders and fitness lifters, *F*(2, 71) = 1.60, *p* = .21. A one-way ANOVA was significant for years lifting, *F*(2, 71) = 4.09, *p* < .05, with expert bodybuilders (*M* = 7.95) and novice bodybuilders (*M* = 7.48) significantly more experienced than fitness lifters (*M* = 3.96). For weekly frequency data, there were also significant group differences, *F*(2, 71) = 6.18, *p* < .05, with expert (*M* = 5.00) and novice (*M* = 5.13) bodybuilders working out significantly more often than fitness lifters (*M* = 3.63). For session time, significant group differences also occurred, *F*(2, 71) = 5.35, *p* < .05, with expert (*M* = 81.73) and novice (*M* = 76.55) bodybuilders spending significantly more time per workout than fitness lifters (*M* = 52.21). Finally, for workout intensity, there was another significant group main effect, *F*(2, 71) = 10.80, *p* < .05, with expert (*M* = 2.58) and novice (*M* = 2.50) bodybuilders working out typically at a moderate-high intensity, and fitness lifters (*M* = 1.84) typically exerting at a light-moderate intensity (see Table 1).

### Exercise Dependence Scale

A significant between-groups result occurred with EDS total score, *F*(2,71) = 4.26, *p* < .05, and Tukey-tests indicated that expert bodybuilders (*M* = 75.19) scored significantly higher than fitness lifters (*M* = 60.42) (see Table 1). The MANOVA group main effect for the seven EDS scales was not significant, Wilks’ lambda = .72, *F*(14,130) = 1.49, *p* = .07, so no further univariate analysis occurred.

A frequency analysis of EDS total scores was undertaken to find the percentage of participants identified by the scale to be “at risk” for ED. A total of 13.5% (*n* = 10) were identified as “at risk”, 82.4% (*n* = 61) as “nondependent symptomatic”, and 4.1% (*n* = 3) as “nondependent asymptomatic”. Of the 10 “at risk” for ED, nine were bodybuilders and one was a fitness lifter.

**Table 1.** Means and standard deviations of lifting type group differences

	ExpBB (*n* = 26)		NovBB (*n* = 29)		FitLif (*n* = 19)		*F*	df	*p*
	*M*	(SD)	*M*	(SD)	*M*	SD)
Total lift time	448.08	(218.24)	441.21	(252.42)	333.42	(224.79)	1.60	(2, 71)	.21
Years lifting	7.95^a^	(5.65)	7.48^b^	(5.23)	3.96^ab^	(3.16)	4.09	(2, 71)	.02
Weekly freq.	5.00^a^	(1.10)	5.13^b^	(1.99)	3.63^ab^	(1.26)	6.18	(2, 71)	.003
Session time	81.73^a^	(30.98)	76.55^b^	(32.16)	52.21^ab^	(19.88)	5.35	(2, 71)	.007
Intensity	2.58^a^	(.51)	2.50^b^	(.51)	1.84^ab^	(.50)	10.80	(2, 71)	.001
Total EDS	75.19^a^	(16.15)	71.41	(19.09)	60.42^a^	(15.11)	4.26	(2, 71)	.02
Bodybuilding Dependence Scale									
Mastery depen.	7.88	(3.33)	8.34	(3.21)	6.17	(3.61)	2.56	(2, 71)	.08
Social depen.	20.92^a^	(5.30)	21.59^b^	(6.61)	12.47^ab^	(4.79)	16.68	(2, 71)	.001
Training depen.	14.46^a^	(2.77)	14.41^b^	(3.91)	9.79^ab^	(3.77)	12.35	(2, 71)	.001
Muscle Dysmorphia Inventory									
Supplement use	18.42^a^	(4.82)	14.10^b^	(6.21)	7.68^ab^	(3.77)	23.43	(2, 71)	.001
Pharmacol. use	4.27	(1.71)	4.34	(2.58)	3.63	(1.64)	.76	(2, 71)	.47
Dietary behavior	23.92^a^	(3.78)	21.44^b^	(5.32)	13.89^ab^	(6.39)	21.80	(2, 71)	.001
Exercise depen.	19.54^a^	(3.64)	16.93^b^	(3.66)	11.31^ab^	(3.93)	27.24	(2, 71)	.001
Physique conc.	13.04	(3.84)	13.97	(7.24)	10.53	(2.98)	15.31	(2, 71)	.10
Size symmetry	17.62^a^	(4.34)	16.17^b^	(6.69)	10.26^ab^	(4.29)	11.09	(2, 71)	.001
Drive for thin	23.54	(7.87)	23.03	(6.93)	24.10	(7.44)	.12	(2, 71)	.89

^a, b^ *p* < .05 (*Notes:* ^a^ indicates significant differences between experienced bodybuilders and fitness lifters; ^b^ indicates significant differences between novice bodybuilders and fitness lifters.)

### Bodybuilding Dependence Scale

A significant overall MANOVA group main effect (Wilks' lambda = .66, *F*(6, 138) = 5.30, *p* < .05) was calculated. Univariate *F*-tests showed that Training Dependence was significant (*F*(2, 71) = 12.35, *p* < .001), and Tukey-tests indicated that expert (*M* = 14.46) and novice (*M* = 14.41) bodybuilders were significantly higher than fitness lifters (*M* = 9.79). A significant Social Dependence scale group main effect (*F*(2, 71) = 16.68, *p* < .001) indicated that expert (*M* = 20.92) and novice (*M* = 21.59) bodybuilders were significantly higher than fitness lifters (*M* = 12.47) (see Table 1). No significant group main effect was found for Mastery Dependence, *F*(2, 71) = 2.56, *p* = .08.

### Muscle Dysmorphia Inventory

A significant overall MANOVA group main effect (Wilks' lambda = .44, *F*(12, 132) = 5.59, *p* < .05) occurred. Univariate *F*-tests indicated significant differences in Supplement Use (*F*(2, 71) = 23.43, *p* < .001), Dietary Behavior (*F*(2, 71) = 21.80, *p* < .001), Exercise Dependence (*F*(2, 71) = 27.24, *p* < .001), and Size Symmetry (*F*(2, 71) = 11.09, *p* < .01). Follow up Tukey post hoc tests showed that expert and novice bodybuilders scored significantly higher than fitness lifters on these four scales (see Table 1).

### Drive for Thinness Scale

No significant differences occurred in DFT score between the lifting groups, *F*(2, 71) = .12, *p* = .89.

## DISCUSSION

In general, bodybuilders spent more years, time in the gym, and worked out harder than fitness lifters. This finding is similar to differences in workout frequency reported for male bodybuilders and fitness lifters (Hale et al., 2010), but as Hausenblas and Symons Downs (2002) reported, exercise behavior and history alone are not adequate predictors of ED. The finding that bodybuilders’ typical workout was moderate-high in intensity compared to fitness lifters' light-moderate intensity was similar to recent findings of Cook, Hausenblas and Rossi (2013), who reported that participants who wanted to gain weight (e.g., bodybuilders) had significantly higher amounts of strenuous exercise than women who wanted to lose weight (e.g., fitness lifters). Other antecedent variables must be examined to try to understand the etiology of the disorder.

The hypothesis that bodybuilders would show higher scores in exercise dependence was partially supported. Although a predicted higher score was calculated for expert bodybuilders over fitness lifters in total EDS, the MANOVA for scale differences just failed to reach significance. Re-examination showed that the statistical power was .87, which is barely adequate for a multivariate analysis involving seven dependent variables and 74 participants. The finding for higher total EDS scores is similar to other previous studies (Hale et al., 2010; Hurst et al., 2000) that have examined differences between different lifting types. More participants should have been measured in each lifting group,

In this sample of female lifters, the prevalence of ‘at-risk’ behavior for ED behaviors (13.5%) was found to be higher than other college-age samples measured by the EDS (3.4%, Hausenblas & Symons Downs, 2002; 3.6–5%, Symons Downs et al., 2004). Past findings (Allegre, Souville, Therme & Griffiths, 2006; Hausenblas & Symons Downs, 2002; Terry, Szabo & Griffiths, 2004) of mixed gender samples have been conservatively in the 3–13% range. In this study nine out of 10 of the “at risk” scores came from bodybuilder groups.

This at-risk finding is further supported by results from the BDS analysis. On two of the three scales, female bodybuilders scored higher than fitness lifters. These findings are supported by Hale et al. (2010) and Smith and Hale (2004) with male bodybuilders and fitness lifters.

The results for the MD assessment also provided partial support for the hypothesis that female novice and expert bodybuilders would score higher. The significant finding on the Exercise Dependence scale suggests that women bodybuilders, like male bodybuilders, are at extremely high risk for MD symptoms (Smith & Hale, 2004; Tod & Lavallee, 2010). These findings further support the qualitative findings of Bolin (1992), Guthrie and Castelnuovo (1992), and Klein (1986, 1992) and quantifiable results of Goldfield (2009) with women bodybuilders. With no differences reported here between novice and experienced bodybuilders, it suggests that women who are attracted to serious bodybuilding programs may arrive with symptoms of MD intact already or may develop these symptoms soon after committing to regimented training.

Finally, the non-significant findings between lifting groups on the Drive for Thinness scale rejected our research hypothesis. Since Hausenblas and Symons Downs' (2002) criteria for secondary exercise dependence is a score of 14 or better, all three groups seem to be highly at risk for an eating disorder. The high scores also suggest that in addition to a potentially high drive for muscularity (McCreary & Sasse, 2000), all female weight lifters may also want to remain extremely lean.

Recently Berczik et al. (2012) have suggested that ED should be more appropriately labeled as exercise addiction with six common symptoms (salience, mood modification, tolerance, withdrawal symptoms, personal conflict, and relapse). Furthermore, they assert that exercise addiction is a form of behavioral addiction because of its preoccupation with the behavior when it is prevented or delayed. It is clear that measurement of ED (addiction?) needs improved diagnostic processes. A recent study by Heaney, Ginty, Carroll and Phillips (2011) showed that ED participants produced a blunted cardiovascular and cortisol reaction to stress, similar to those seen in alcohol and smoking dependence; this finding may offer a more accurate, future objective measurement.

This study contains several design limitations. It was a correlational, cross-sectional design that could not provide cause-and-effect findings which might help predict the etiology of ED and MD. In addition, the sample was voluntary and limited to a small group of bodybuilders from one major competition and several health clubs with a non-random fitness group used as a comparator, which reduced statistical power for internal validity and decreased the potential for external validity. The questionnaires selected are only indicative of ‘at risk’ symptoms inherent in ED and MD; only clinical diagnostic procedures combined with questionnaires and possible biochemical analyzes can lead to clear diagnosis. Future research needs more diverse samples of female weight lifters and ED and MD self-report measures given in random order that include measures that control for social desirability.

In conclusion, the overall findings of this study support the hypothesis that female bodybuilders, whether new or experienced competitors, show the same high risks for ED and MD that male bodybuilders have shown (Hale & Smith, 2012; Tod & Lavallee, 2010). This study is one of the first quantifiable cross-sectional designs to compare differences in potentially pathological exercise behaviors and eating disorders in different female weight lifting groups. The findings suggest that either gender of bodybuilders may be at high risk for potentially addictive behavioral disorders (exercise dependence, muscle dysmorphia).
